# The redox-active defensive Selenoprotein T as a novel stress sensor protein playing a key role in the pathophysiology of heart failure

**DOI:** 10.1186/s12967-024-05192-w

**Published:** 2024-04-20

**Authors:** Anna De Bartolo, Teresa Pasqua, Naomi Romeo, Vittoria Rago, Ida Perrotta, Francesca Giordano, Maria Concetta Granieri, Alessandro Marrone, Rosa Mazza, Maria Carmela Cerra, Benjamin Lefranc, Jérôme Leprince, Youssef Anouar, Tommaso Angelone, Carmine Rocca

**Affiliations:** 1 Cellular and Molecular Cardiovascular Pathophysiology Laboratory, Department of Biology, E. and E. S. (DiBEST), University of Calabria, Arcavacata di Rende, 87036 Cosenza, Italy; 2 Department of Health Science, University Magna Graecia of Catanzaro, 88100 Catanzaro, Italy; 3 Department of Pharmacy, Health and Nutritional Sciences, University of Calabria, 87036 Rende, Italy; 4 Centre for Microscopy and Microanalysis (CM2), Department of Biology, E. and E. S. (DiBEST), University of Calabria, 87036 Rende, Italy; 5 Organ and System Physiology Laboratory, Department of Biology, E. and E. S. (DiBEST), University of Calabria, Arcavacata di Rende, 87036 Cosenza, Italy; 6 UNIROUEN, Inserm U1239, Neuroendocrine, Endocrine and Germinal Differentiation and Communication (NorDiC), Rouen Normandie University, 76000 Mont-Saint-Aignan, France; 7 UNIROUEN, UMS-UAR HERACLES, PRIMACEN, Cell Imaging Platform of Normandy, Institute for Research and Innovation in Biomedicine (IRIB), 76183 Rouen, France; 8 National Institute of Cardiovascular Research (INRC), 40126 Bologna, Italy

**Keywords:** Antioxidants, Cardiac dysfunction, Cardiomyocytes, Peptides, Selenoproteins

## Abstract

Maladaptive cardiac hypertrophy contributes to the development of heart failure (HF). The oxidoreductase Selenoprotein T (SELENOT) emerged as a key regulator during rat cardiogenesis and acute cardiac protection. However, its action in chronic settings of cardiac dysfunction is not understood. Here, we investigated the role of SELENOT in the pathophysiology of HF: (i) by designing a small peptide (PSELT), recapitulating SELENOT activity via the redox site, and assessed its beneficial action in a preclinical model of HF [aged spontaneously hypertensive heart failure (SHHF) rats] and against isoproterenol (ISO)-induced hypertrophy in rat ventricular H9c2 and adult human AC16 cardiomyocytes; (ii) by evaluating the SELENOT intra-cardiomyocyte production and secretion under hypertrophied stimulation. Results showed that PSELT attenuated systemic inflammation, lipopolysaccharide (LPS)-induced macrophage M1 polarization, myocardial injury, and the severe ultrastructural alterations, while counteracting key mediators of cardiac fibrosis, aging, and DNA damage and restoring desmin downregulation and SELENOT upregulation in the failing hearts. In the hemodynamic assessment, PSELT improved the contractile impairment at baseline and following ischemia/reperfusion injury, and reduced infarct size in normal and failing hearts. At cellular level, PSELT counteracted ISO-mediated hypertrophy and ultrastructural alterations through its redox motif, while mitigating ISO-triggered SELENOT intracellular production and secretion, a phenomenon that presumably reflects the extent of cell damage. Altogether, these results indicate that SELENOT could represent a novel sensor of hypertrophied cardiomyocytes and a potential PSELT-based new therapeutic approach in myocardial hypertrophy and HF.

**Graphical Abstract:**

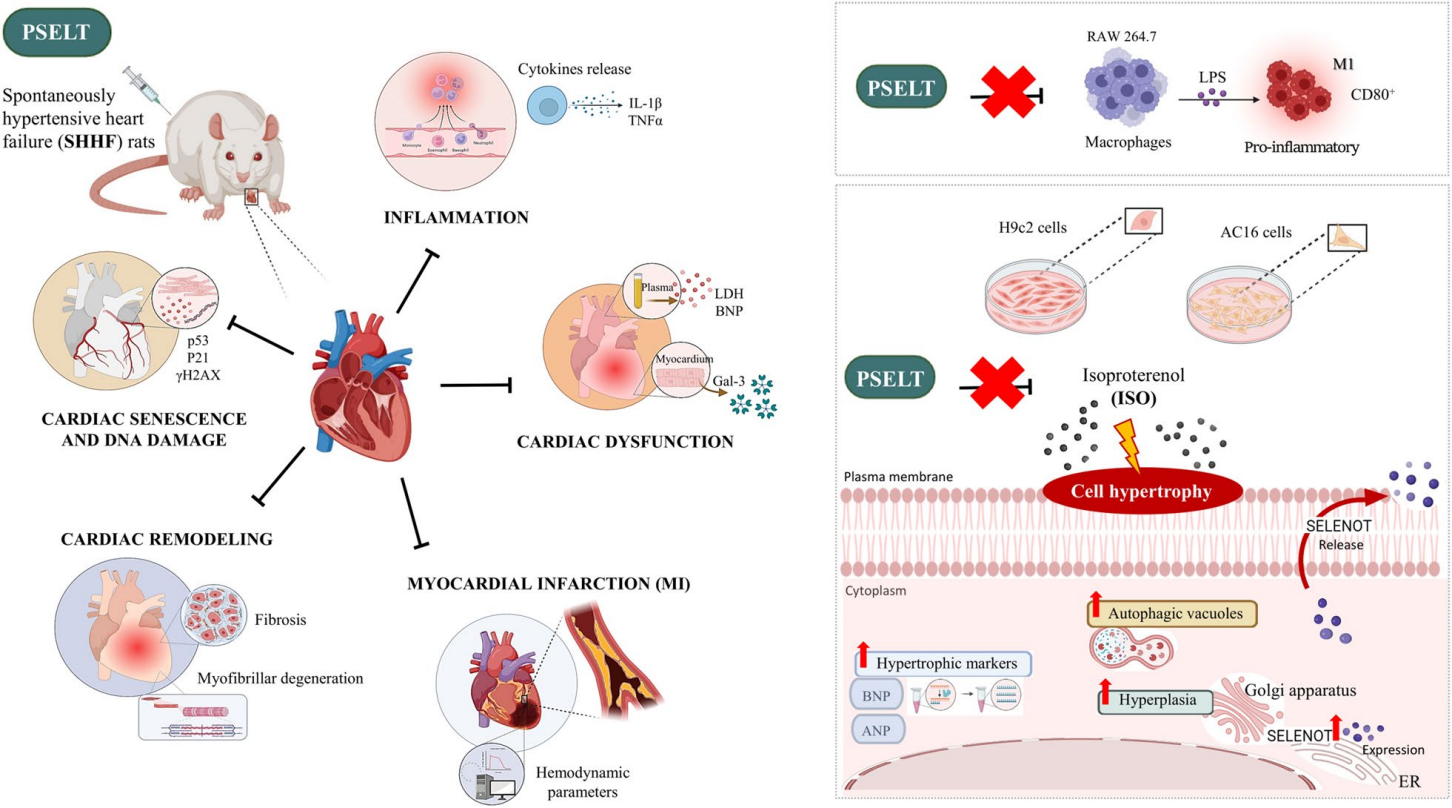

## Introduction

Heart failure (HF) represents a highly prevalent health condition that occurs when the heart, pumping an inadequate blood volume, progressively becomes unable to supply the metabolic needs of the tissues. Even if hypertension and hypertension-induced hypertrophy are among the most recognized risk factors, it is currently admitted that HF consists of multifactorial and complex changes in cardiovascular physiology [1, 2]. Thus, despite the arsenal of therapies proposed, the affected population increases year by year and the management in clinical practice is even more complicated [3]. Indeed, expenses related to HF are a burden for healthcare systems worldwide since high hospitalization rates, secondary to the associated morbidity and mortality, are recorded [4]. In addition, as in the case of other important cardiac disorders, such as hypertrophic cardiomyopathy (HCM), sudden cardiac death is the most deleterious complication of this pathological state [5–7].

It is widely accepted that myocardial infarction (MI) remains the most frequent cause of HF [1, 8]. MI culminates in the necrosis of cardiac cells, secondary to a reduced oxygen supply that, in turn, causes deleterious changes in their bioenergetics [9]. The acute handling of MI operated by a rapid revascularization is associated with reduced mortality rates but, on the other hand, the detrimental effects induced by acute myocardial ischemia/reperfusion injury (MI/R), can increase the risk of arrhythmias and HF [10–12]. A role for the β-adrenergic signaling system has also been postulated [13]. However, despite increased circulating catecholamine (CA) levels, HF patients show reduced β-adrenergic receptor (β-AR) density and responsiveness [14] due to G protein alterations, decreased adenylyl cyclase activity and functional uncoupling of β-AR driven by an overactivation of β-AR kinase 1 (βARK1) [14].

From a clinical point of view, the current therapy for HF is based on the use of angiotensin/aldosterone receptor antagonists, angiotensin-converting enzyme inhibitors (ACE-I), diuretics, β-AR blockers and/or calcium channel inhibitors [15]. These treatments mitigate all the related symptoms, ameliorating the quality of life, but none of them have demonstrated a favourable impact on survival outcome of HF patients. Therefore, new directions in the field of HF research and clinical treatment are constantly under investigation, as novel therapeutic options are urgently needed.

Selenoproteins are a family of potent endogenous antioxidant proteins harbouring the rare amino acid selenocysteine (Sec) to act as the first line of defence against oxidants [16]. Selenoprotein T (SELENOT), one of the most important members of this family, is a crucial redox-active protective protein located in the endoplasmic reticulum (ER). It is involved in maintaining ER homeostasis—with a key role in protein folding and maturation—and redox circuits through its oxidoreductase activity [17, 18]. As an essential ER protein, SELENOT is strongly expressed in the heart ER during early cardiogenesis in rat, where it is required during early hyperplastic growth of cardiomyocytes [19]. The decline in SELENOT cardiac expression during rat postnatal and adult physiological stages, together with its reactivation following cardiac stress in the mature heart, indicate that this selenoprotein is essential not only for cardiac tissue differentiation but it is also recruited to protect the heart from ischemia-induced oxidative burst, thus acting as a potential redox-sensing protein [19, 20]. Accordingly, upon cardiac/endothelial stressful conditions, like ischemic insult, lipotoxicity, lipopolysaccharide (LPS), and unfolded protein response associated to ER stress, SELENOT becomes indispensable to pre- or post-condition the heart by counteracting ER stress, proteotoxicity, oxidative stress, and apoptosis (*i.e.,* major events contributing to vascular and cardiac diseases, including HF) [19, 21–23].

Given the key involvement of SELENOT in the heart physiology and pathophysiology, reflected by its in vitro and acute ex vivo protective profile, here we evaluated the chronic cardioprotective role of SELENOT in a rat model of HF. In particular, and to move toward translatability, we took advantage of a small mimetic SELENOT peptide, named PSELT, recognized for its capability of recapitulating the activity of the full-length protein through the redox CVSU active motif of SELENOT, and tested its beneficial action in SHHF (spontaneously hypertensive, heart failure-prone rats), rat cardiomyocytes, and human ventricular cardiomyocytes. We also evaluated the SELENOT intra-cardiomyocyte production and secretion under hypertrophied stimulation.

## Materials and methods

### Peptides and drugs

PSELT, the SELENOT-mimetic peptide [SELENOT_43–52_ (H–Phe–Gln–Ile–Cys–Val–Ser–Sec–Gly–Tyr–Arg–OH)] and its inactive form, indicated as inert-PSELT [I-PSELT (Ser 46,49)], were chemically synthesized by automated peptide synthesizer (CEM, Saclay, France) as previously reported [19]. Dulbecco’s Modified Eagle Medium F-12 (DMEM/F-12), Dulbecco’s Modified Eagle Medium (DMEM), Dulbecco’s phosphate buffer saline (DPBS), penicillin/streptomycin (P/S), L-Glutamine, UltraPure™ DNase/RNase-Free Distilled Water, Lipofectamine^®^ 2000 Reagent were from Thermo Fisher Scientific (Waltham, MA, USA). Foetal bovine serum (FBS) and 0.25% Trypsin–EDTA were from Corning (New York, USA). Bovine serum albumin (BSA), and non-fat dried milk were from PanReac AppliChem (Glenview, IL, USA). KCl, NaCl, NaHCO_3_, CaCl_2_, MgSO_4_, KH_2_PO_4_, NaH_2_PO_4_, Na_2_HPO_4_, mannitol, glucose, Na-pyruvate, β-nicotinamide adenine dinucleotide (NADH), reduced disodium salt hydrate, 2,4 dinitrophenylhydrazine (DNPH), diethyl ether, ethylenediaminetetraacetic acid (disodium salt), diethylenetriamine pentaacetic acid, pyrogallol, streptomycin sulfate, tween-20, isoproterenol, 4',6-diamidino-2-phenylindole (DAPI) were from Sigma Aldrich (Saint Louis, MO, USA). Absolute ethanol and hydrochloric acid were from Carlo Erba Reagents (Cornaredo, Milan, Italy). Before each experiment, all solutions were freshly prepared.

### Animals

Experimental procedures were carried out using male healthy control Wistar (WST) rats and spontaneously hypertensive heart failure (SHHF) rats, a congenital model of dilated cardiomyopathy with hypertension progressing to HF [24] (Charles River laboratories, Milan-Italy). Animals were individually housed in cages under controlled light (12 h light/dark cycle), temperature (23–25 °C) and humidity (50–55%) and fed ad libitum with standard diet (Envigo, Udine-Italy). The study was conducted in accordance with the Declaration of Helsinki, the Italian law (D.L. 26/2014), the Guide for the Care and Use of Laboratory Animals [U.S. National Institutes of Health (NIH), Bethesda, MD, USA] and the Directive 2010/63/EU of the European Parliament on the protection of animals used for science. The project was approved by the Italian Ministry of Health, Rome, and the ethics review board.

#### In vivo* study: experimental groups*

In vivo treatments in rats started at the 15th month of age, time at which SHHF animals display significant loss of cardiac function, together with biochemical, structural, and hemodynamic alterations denoting a mid/late-stage of HF, as widely reported [24, 25]. Both WST and SHHF rats were intraperitoneally (i.p.) treated with saline (NaCl 0.9%) or PSELT (80 µg/Kg) every three days for eighteen days according to a similar protocol adopted by Yashiro et al., [26]. This dose of PSELT (corresponding to ~ 65 nmol/kg) is similar to the PSELT nanomolar concentrations able to exert cardioprotective action, as indicated in our previous publications [19, 21, 22]. Depending on saline or PSELT administration, WST and SHHF rats were divided in the following 4 groups: (i) WST + saline (WST, n = 6); (ii) WST + PSELT (WST + PSELT, n = 6); (iii) SHHF + saline (SHHF, n = 6); (iv) SHHF + PSELT (SHHF + PSELT, n = 6).

At the end of the in vivo protocols, animals were anesthetized with i.p. injection of ethyl carbamate (2 g/kg body weight), and then sacrificed. In particular, for biochemical evaluation, blood samples were collected with heparinized syringes and plasma was separated by centrifugation at 3000*g* (15 min, 4 °C). Plasma samples were used for the quantification of interleukin-1β (IL-1β), tumor necrosis factor-α (TNF-α), brain natriuretic peptide (BNP), and lactate dehydrogenase (LDH). Cardiac tissues were used to detect galectin-3 (GAL-3), evaluate the ultrastructure and molecular signaling, and assess performance ex vivo.

#### Enzyme-linked immunosorbent assays (ELISAs)

Plasma levels of IL-1β, TNF-α, and BNP were detected by ELISAs provided by Elabscience Biotechnology Inc. USA (IL-1β: E-EL-R0012; TNF-α: E-EL-R2856; BNP: E-EL-R0126) according to the manufacturer’s instructions and as previously described. Cardiac levels of GAL-3 were quantified by ELISA assay from MyBioSource, San Diego, USA (MBS761093) San Diego, United States according to the manufacturer’s instructions.

#### Lactate dehydrogenase (LDH) activity

The enzymatic activity of LDH in plasma samples was spectrophotometrically evaluated according to McQueen method [27] and as previously reported [28, 29]. Enzyme activity, expressed as IU/L, was determined by monitoring the absorbance decrease at 340 nm resulting from NADH oxidation.

#### Transmission electron microscopy (TEM) analysis on cardiac sections

After in vivo treatments, cardiac tissues were fixed in 2.5% glutaraldehyde (in 0.1 M phosphate buffer pH 7.4) for 2 h at 4 °C. Then, three washes were carried out in order to remove residual fixative, and post-fixation was performed in 1% osmium tetroxide (in 0.1 M phosphate buffer pH 7.4) for 2 h at 4 °C. Samples were washed 3 times with phosphate buffer, gradually dehydrated using increasing concentrations of acetone and then embedded in Araldite (Fluka, 10,951). Ultrathin sections by using an RMC Power Tome series ultramicrotome and a diatome diamond knife were prepared and collected on copper grids (EMS, G 300 Cu) to be examined with a Jeol JEM 1400 Plus electron microscope (Peabody, Massachusetts, USA) at 80 kV.

#### Western blot analysis on cardiac tissues

The apex of LV of the hearts deriving from each experimental group was homogenized in ice-cold RIPA lysis buffer supplemented with a mixture of protease and phosphatase inhibitors and centrifuged at 15,000*g* for 20 min at 4 °C, as detailed in previous studies [18, 19, 21]. For insoluble fraction assessment, LV was homogenized in urea-thiourea buffer [7 M urea, 2 M thiourea, and 4% (w/v) CHAPS, in 30 mM Tris–HCl (pH 8.5)] [30] supplemented with protease and phosphatase inhibitors and centrifuged at 15,000*g* for 15 min at 4 °C. The supernatant was collected, and the protein concentration was determined by Bradford assay. Equal amounts of proteins (30–80 μg) were separated on 10% SDS-PAGE gels for desmin and p53, on 12% SDS-PAGE gels for SELENOT, CTGF, and p21, and on 15% SDS-PAGE for phosphorylated gamma-H2A histone family member X (γH2AX, H2AX Ser139 phosphorylation) and transferred to nitrocellulose membrane by trans-blot turbo system (Bio-Rad). Membranes were blocked in 5% non-fat dried milk at room temperature for 1 h, washed three times with tris-buffered saline containing 0.1% Tween 20 (TBST) and incubated overnight at 4 °C with the specific primary antibodies diluted 1:3000 [desmin (PA5-16,705)], 1:500 [p21 (SC-6246)], 1:1000 [CTGF (TA806803), p53 (sc-126), and SELENOT (LS-C168948)] in TBST containing 5% bovine serum albumin (BSA) or TBST containing 1% non-fat dried milk (γH2AX, 05–636, final concentration 0.5 µg/ml). β-actin antibody (1:1000) (SC-47778 C4) was used as loading control. Then, membranes were incubated with peroxidase-linked secondary antibodies at room temperature for 1 h (anti-rabbit and anti-mouse diluted 1:2000 and 1:1000, respectively). Immunodetection was performed using Clarity Western ECL Substrate (Bio-rad). Bands densitometry was evaluated by measuring area and pixel intensity using ImageJ 1.6 software (National Institutes of Health, Bethesda, MD, USA), as previously indicated [18, 19, 21].

#### Measurement of matrix metalloproteinases (MMPs) activity by zymography

The enzymatic activity of MMPs was assessed by SDS-PAGE zymography using gelatin as substrate. An equal amount of proteins for heart samples (60 µg) was loaded under non-denaturing conditions on 8% SDS-PAGE gels containing 0.1% gelatin. Once electrophoresis was completed, the gels were washed for 30 min with buffer I [Tris–HCl (pH 7.5) and 2.5% Triton X-100], and then incubated overnight at 37 °C in buffer II [150 mM NaCl, 5 mM CaCl_2_, 50 mM Tris–HCl (pH 7.6)]. Finally, the gels were stained with 2% Coomassie Brilliant Blue R250 (Sigma Aldrich), 25% methanol, 10% acetic acid and then de-stained in 2% methanol–4% acetic acid for 1 h [31]. The gelatin digestion areas by MMPs were visualized and quantified with ImageJ 1.6 software. MMP-2 activity was expressed as a percentage of total band area in the 4 experimental groups.

### Ex vivo study: Langendorff isolated rat heart perfusion

At the end of in vivo procedures and after anaesthesia, chest was opened and heart excised in order to be connected to the Langendorff apparatus under a retrograde perfusion with Krebs–Henseleit (KH) solution at a constant flow rate of 12 mL/min, 37 °C, as reported in previous studies [18, 19, 21, 32, 33]. Hemodynamic parameters were recorded every 10 min and analysed using PowerLab data acquisition system (AD Instruments, Sydney, New South Wales, Australia).

#### Basal conditions

Basal cardiac performance was evaluated by monitoring inotropism, in terms of developed left ventricle (LV) pressure (dLVP; mmHg, index of contractile activity) and maximal value of the first LVP derivative [+ (LVdP/dT) max; in mmHg/s, index of maximal LV contraction rate], lusitropism, assessed through the maximal rate of LVP decline [− (LVdP/dT) max; mmHg/s], and LV end diastolic pressure (LVEDP; mmHg), coronary vasomotility, assessed by monitoring coronary pressure (CP; mmHg), and heart rate (HR) changes (beats/min) used to evaluate chronotropism.

#### Ischemia/Reperfusion (I/R) protocols

Hearts of each experimental group were subjected to I/R protocols in order to reproduce I/R injury (IRI). After 40 min stabilization, hearts were subjected to 30 min, zero-flow global ischaemia followed by 120 min of reperfusion. Post-ischemic cardiac recovery was assessed by monitoring post-ischemic dLVP and LVEDP, being this latter an index of contracture, defined as its increase of 4 mmHg above the baseline level [18, 19, 21, 34, 35]. The stability of all preparations was evaluated by measuring cardiac performance every 10 min and these parameters were stable up to 190 min. Accordingly, sham hearts were perfused with KH buffer for 190 min.

#### Infarct size (IS) evaluation

Immediately after IRI protocols, hearts were rapidly removed from the perfusion apparatus to assess infarct area by nitro-blue tetrazolium staining in a blinded fashion manner, as detailed in our previous studies [18, 19, 21]. Unstained necrotic tissues were carefully separated from stained viable tissues to calculate IS, expressed as a percentage of total LV mass.

### In vitro* study in murine macrophages and rat and human cardiomyocytes: cell cultures*

Murine macrophages RAW 264.7 cell line (ATCC, Cat# TIB-71) were cultured in DMEM (4.5 g/L glucose) (Gibco) containing 10% FBS, 2 mM glutamine, 1% P/S and incubated at 37 °C in a humidified chamber, 5% CO_2_. For experiments RAW 264.7 cells were seeded in 6-well plates for 24 h in a humidified atmosphere, 5% CO_2_ at 37 °C. H9c2 cardiomyoblast cells (ATCC, Cat# CRL-1446) were cultured in DMEM/F-12 (Gibco) supplemented with 10% FBS, 1% P/S (Thermo Fisher Scientific) and incubated at 37 °C in a humidified chamber containing 5% CO_2_. For experiments cells were seeded in complete medium and incubated for 48 h at 37 °C, 5% CO_2_, as previously reported [21, 22, 36]. AC16 human cardiomyocytes (Millipore-Sigma Aldrich, Cat. # SCC109) were grown in DMEM/F-12 supplemented with 12.5% FBS, 2 mM glutamine and 1% P/S, and incubated in a humidified atmosphere of 95% air and 5% CO_2_ at 37 °C. For experiments, cells were plated in complete medium for 24 h at 37 °C, 5% CO_2_, as previously reported [37, 38].

#### Analysis of CD80 by flow cytometry in RAW 264.7 macrophages

RAW 264.7 cells were seeded 6 well plates and treated with vehicle (saline, NaCl 0.9%) (CTRL) or LPS (100 ng/ml) [39, 40] and PSELT (5 nM) alone or in co-treatment for 24 h. At the end of the treatments, cells were washed with cold DPBS detached with trypsin–EDTA and centrifuged. The pellet was resuspended in 100 µl of cold DPBS containing FITC anti-CD80 (B7-1) monoclonal antibody (11–0801-82) (Thermo Fisher Scientific) according to the manufacturer's instructions. After 30 min incubation at 4 °C, RAW 264.7 cells were washed with DPBS (1X) and centrifuged at 500 g for 5 min, then re-suspended in DPBS and analyzed by flow cytometry (CytoFLEX Beckman, Beckman Coulter, Milan, Italy). Data analysis was performed using CytExpert Beckman Coulter software (Beckman Coulter, Milan, Italy).

#### Morphological staining in H9c2 cardiomyoblast cells

Morphological changes were assessed by May-Grunwald Giemsa (MGG) staining, as previously reported [36, 41]. H9c2 cells were seeded in 60 mm culture dishes, treated with vehicle (saline, NaCl 0.9%), isoproterenol (ISO) (100 µM) [42], ISO + PSELT (5 nM) or PSELT alone [22], while another set of H9c2 cells were treated with vehicle, ISO, ISO + I-PSELT (5 nM) or I-PSELT alone, and incubated in a humidified atmosphere at 37 °C for 48 h. After MGG staining, H9c2 cardiomyocytes were visualized by using Olympus BX41 microscope, and the images were taken with CSV1.14 software, using a CAM XC-30 for image acquisition. Cell-surface area was analyzed by using Image J 1.6 (NIH). Data were expressed as percentage of a relative increase in cell-surface area.

#### Gene silencing of endogenous SELENOT by small interfering RNA (siRNA) in H9c2 cardiomyocytes

Gene silencing for SELENOT was performed in H9c2 cardiomyocytes, as previously described [21, 22]. Briefly, cells were seeded in 60 mm cell culture dishes and incubated for 48 h at 37 °C, 5% CO_2_. SELENOT siRNA (100 nM) was transfected into H9c2 cells using Lipofectamine 2000 reagent following the manufacturer’s instructions (Invitrogen). Negative control si-RNA (si-NC) was used to evaluate sequence-specific silencing from non-specific effects. Both control siRNA-A and siRNA for SELENOT were purchased from Santa Cruz Biotechnology. H9c2 cardiomyocytes were transfected in serum-free medium for 6 h; then, medium was replaced with full media and cells were incubated for 36 h at 37 °C, 5% CO_2_. Cells were treated with vehicle or ISO (100 µM) for 48 h and, at the end of the treatments, cell morphology was evaluated by MGG staining, as described above. Data were expressed as percentage of relative increase in cell-surface area.

#### Western blot analysis on H9c2 cardiomyocytes

H9c2 cells treated with vehicle, ISO (100 μM), ISO + PSELT (5 nM) and PSELT alone were processed as reported previously [22, 36, 41]. After establishing protein concentration in the supernatant by Bradford assay, 50 μg of proteins were loaded on SDS-PAGE gel, then electroblotted onto a nitrocellulose membrane and probed with the primary specific antibody against SELENOT. Antibody against β-actin was used as loading control. Following the incubation with the primary antibodies, membranes were incubated with peroxidase-conjugated secondary antibodies at room temperature for 1 h (anti-rabbit and anti-mouse diluted 1:2000 and 1:1000, respectively). Immunodetection and densitometric analysis were performed as described above.

#### Gene expression assessment by qPCR

H9c2 and AC16 cardiomyocytes were grown in 6-well plates to reach 70–80% confluence and treated with vehicle (CTRL), ISO (100 μM), ISO + PSELT, and PSELT (5 nM) alone for 48 h. At the end of treatments, total RNA was isolated using TRIzol reagent (Invitrogen) according to the manufacturer’s protocol. 2 μg of total RNA were reverse transcribed to provide cDNA using High-Capacity cDNA Reverse Transcription Kit (Applied Biosystems™, Thermo Fisher Scientific). cDNA was amplified by qPCR using SYBR™ Select Master Mix (Thermo Fisher Scientific) according to the manufacturer’s instructions on QuantStudio™ 1 Real-Time PCR System apparatus (Thermo Fisher Scientific). Samples were analyzed in duplicate (n = 3 independent experiments). 18S rRNA was used as an internal control. The primers used for the amplification were listed in Table 1. Relative gene expression levels were calculated using the 2^−ΔΔCt^ method [43, 44].

**Table 1:** Primer sequences used for qPCR amplification

Gene	GenBank ID	Forword primer 5′–3′	Reverse primer 5′–3′
*Rat_ NppA (ANP)*	NM_012612.2	GGAAGTCAACCCGTCTCAGA	TGGGCTCCAATCCTGTCAAT
*Rat_ NppB (BNP)*	NM_031545.1	CCAGAACAATCCACGATGCA	GCAGCTTGAACTATGTGCCA
*Rat_ 18 s rRNA*	NR_046237.2	CATTCGAACGTCTGCCCTAT	GTTTCTCAGGCTCCCTCTCC
*Human_ NPPA (ANP)*	NM_006172.4	CAGCAAGCAGTGGATTGCTCCT	TCTGCGTTGGACACGGCATTGT
*Human_ NPPB (BNP)*	NM_002521.3	GCTGCTTTGGGAGGAAGATG	ATGAGTCACTTCAAAGGCGG
*Human_ 18 s rRNA*	M10098.1	CCCACTCCTCCACCTTTGAC	TGTTGCTGTAGCCAAATTCGTT

#### Assessment of cell-surface area in AC16 human cardiomyocytes

Cellular hypertrophy in AC16 cardiomyocytes was evaluated by Alexa Fluor 568–conjugated phalloidin (Thermo Fisher Scientific) following the manufacturer's instructions (Thermo Fisher Scientific). In brief, cells were seeded on coverslip and incubated for 24 h in humidified chamber 37 °C, 5% CO_2_ and then exposed to vehicle (CTRL), ISO (100 µM), ISO + PSELT, PSELT (5 nM) for 48 h. After treatments, cells were fixed with 3.7% paraformaldehyde in DPBS for 10 min at RT, then permeabilized with 0.1% Triton-X100 for 5 min and incubated with 1% BSA in DPBS for 30 min [45]. Cells were stained with Alexa Fluor 568–conjugated phalloidin for 20 min and then nuclei were counterstained with DAPI. Images were taken CSV1.14 software, using a CAM XC-30 for image acquisition. The cell surface area from 3 independent fields was measured with ImageJ software.

#### Quantitative detection of intracellular and extracellular levels of SELENOT in AC16 human cardiomyocytes

AC16 cells were treated with vehicle (CTRL), ISO (100 μM), ISO + PSELT, and PSELT (5 nM) alone for 24, 48, 72, and 96 h. At the end of each treatment, intra-cardiomyocyte and extracellular levels of SELENOT were quantified in cell extracts and cell supernatants, respectively, by using a “Human Selenoprotein T ELISA” sandwich kit provided by MyBioSource (Cat. No MBS166821) according to the manufacturer’s instructions.

#### Transmission electron microscopy (TEM) analysis on human cardiomyocytes

AC16 human cardiomyocytes exposed to vehicle (CTRL), isoproterenol (ISO) (100 µM), ISO + PSELT (5 nM), PSELT for 48 h, were fixed with 3% glutaraldehyde in 0.1 M phosphate buffer overnight at 4 °C. Post-fixation proceeded in buffered osmium tetroxide, followed by dehydration in a graded acetone series, as previously described [22]. Ultrathin Sects. (60–90 nm in thickness) were cut with a diamond knife, mounted on copper grids (G300 Cu), and imaged using a Jeol JEM 1400-Plus electron microscope operating at 80 kV.

#### Statistical analysis

Data were expressed as means ± SEM. A two-way ANOVA and nonparametric Bonferroni’s multiple comparison test (for post-hoc ANOVA comparisons) were used for hemodynamic analyses. A one-way ANOVA and nonparametric Newman–Keuls multiple comparison test (for post- hoc ANOVA comparisons), were used for all the other analyses. Values for *p < 0.05, **p < 0.01, ***p < 0.001 were considered statistically significant. Statistical analysis was conducted using Prism 5 (GraphPad Software, La Jolla, CA, USA).

## Results

### PSELT relieves systemic inflammation, lipopolysaccharide (LPS)-induced macrophage M1 polarization, and cardiac dysfunction in SHHF rat model

**Fig. 1:**
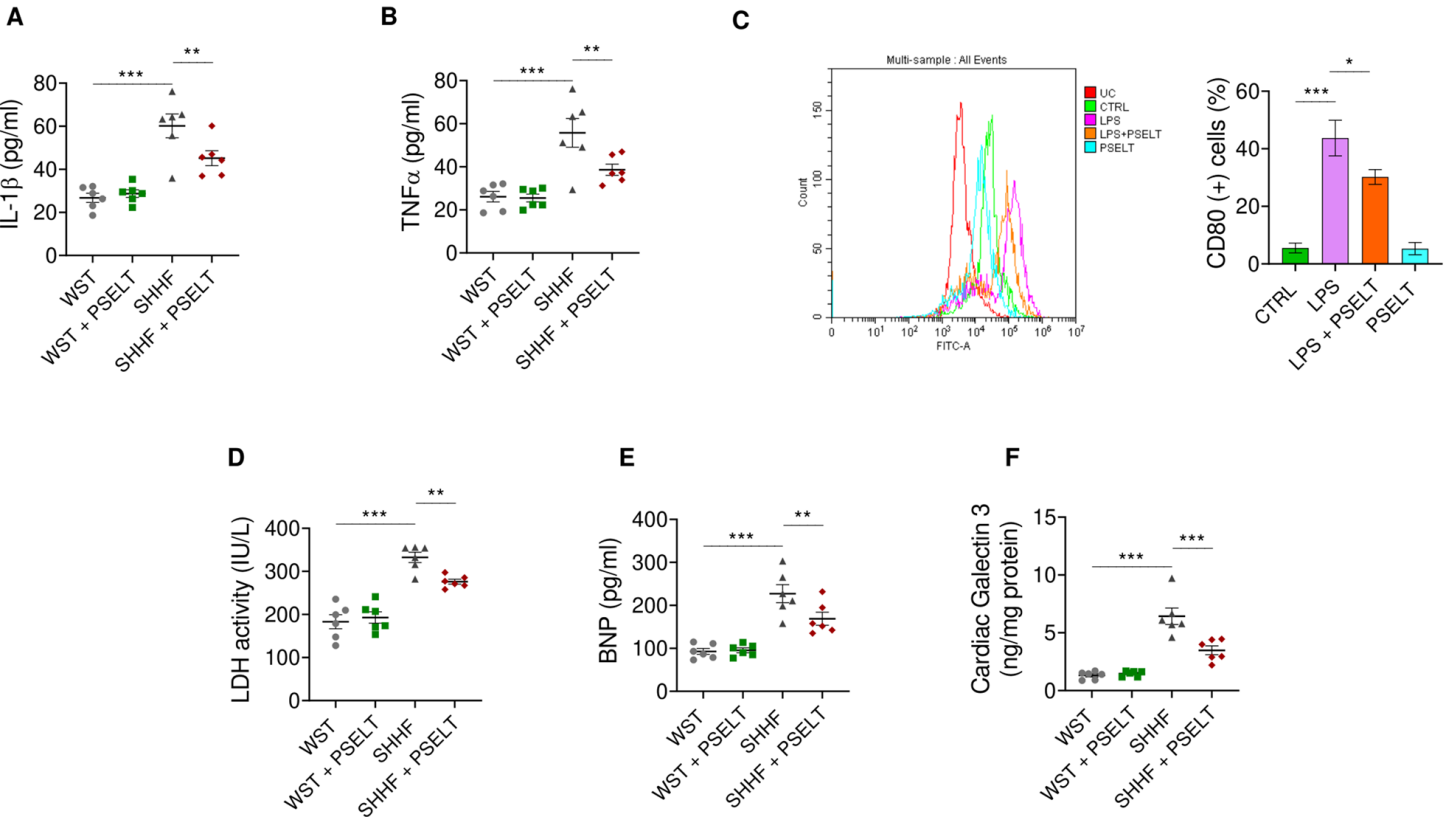
Effects of PSELT on systemic inflammation and cardiac dysfunction. Plasma levels of **A** IL-1β and **B** TNF-α. **C** Flow cytometry analysis of M1 polarization marker CD80 in RAW 264.7 macrophages untreated (unstained cells, UC) or treated with vehicle (CTRL), LPS (100 ng/ml), LPS + PSELT and PSELT (5 nM) for 24 h. Histograms represent the percentages of CD80 positive cells (n = 4 independent experiments). Plasma levels of **D** LDH and **E** BNP, and cardiac levels of **F** Gal-3 (n = 6) in WST, WST + PSELT, SHHF, SHHF + PSELT groups. Data are expressed as means ± SEM. Significant differences were detected by one-way ANOVA and Newman-Keuls multiple comparison test. *p < 0.05; **p < 0.01; ***p < 0.001

In evaluating the in vivo beneficial action of PSELT in SHHF rat model, we first assessed its effect on systemic inflammation by analyzing the plasma levels of the pro-inflammatory cytokines IL-1β and TNF-α. Results showed a significant increase in plasma IL-1β (Fig. 1A) and TNF-α (Fig. 1B) in SHHF rats compared with WST animals (used as healthy controls), and a significant reduction in the levels of these cytokines in SHHF rats treated with PSELT (SHHF + PSELT group) compared with SHHF rats treated with saline (SHHF group). No significant differences were observed between WST and WST + PSELT experimental groups. To investigate the direct anti-inflammatory potential of PSELT, we tested the action of the peptide on LPS-induced macrophage M1 polarization. Results depicted in Fig. 1C indicate that RAW 264.7 cells stimulated with 100 ng/ml of LPS exhibited a significant increase of CD80 expression compared with control cells, while PSELT significantly reduced CD80 expression during LPS stimulation. No differences were detected between control and PSELT alone experimental groups (Fig. 1C). In assessing the in vivo beneficial action of PSELT in SHHF animals, we then investigated whether the peptide also mitigates cardiac dysfunction by analyzing the levels of key markers of HF, such as circulating LDH and BNP, and myocardial GAL-3. We found that the treatment with PSELT mitigated the elevation of plasma LDH and BNP and of cardiac GAL-3 observed in SHHF group compared to WST group. This was revealed by the significant decrease of all the three markers in SHHF + PSELT group compared to SHHF group (Fig. 1D–F). No significant difference has been found between WST and WST + PSELT groups (Fig. 1D–F).

### PSELT ameliorates ultrastructural cardiac alterations and recovers desmin levels in SHHF rat hearts

**Fig. 2:**
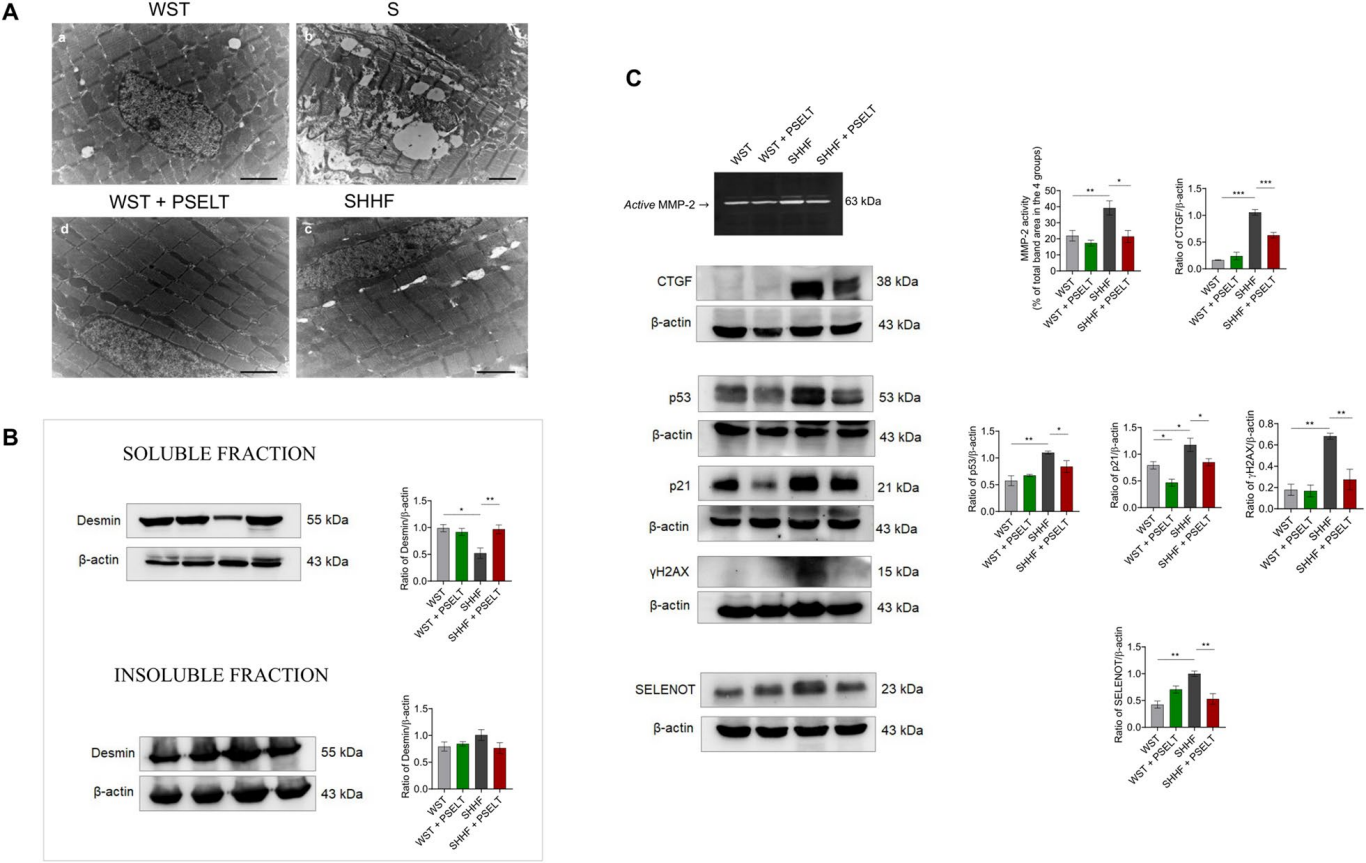
Effect of PSELT on cardiac ultrastructure and on protein expression of SELENOT and fibrosis/senescence-related markers in cardiac tissues. **A** Ultrastructure representations obtained by transmission electron microscopy (TEM) on cardiac sections of control animals (WST) and SHHF animals chronically treated with or without PSELT (scale bar: 2 μm). **B** Western blot analysis of desmin in soluble and insoluble fractions of WST, WST + PSELT, SHHF, SHHF + PSELT hearts. **C** Gelatin SDS-PAGE zymographic analysis of MMP-2 activity in myocardial tissues. Western blot analysis of CTGF, p53, p21, γH2AX, SELENOT, in the hearts of WST, WST + PSELT, SHHF, SHHF + PSELT groups (n = 3). Histograms represent the ratio of densitometric analysis of protein:loading control. Data are expressed as the mean ± SEM (n = 3 independent experiments). Significant differences were detected by one-way ANOVA and Newman-Keuls multiple comparison test. *p < 0.05; **p < 0.01, ***p < 0.001

To study the protective action of PSELT on cardiac ultrastructure, we performed a transmission electron microscopy (TEM) analysis on heart sections of WST and SHHF rats after in vivo treatment with PSELT or saline. Our data revealed that control hearts (WST group) possess the characteristic transverse banding typical of sarcomere organization (formed by thick, darker filaments and thin, lighter filaments) and a single nucleus located in a central position. WST heart also showed lipid droplets and numerous aligned mitochondria to form longitudinal rows that interrupt the continuity of the filaments (Fig. 2Aa). Conversely, many alterations seen in the heart of SHHF rats were related to a marked disorganization of the spatial orientation of the myofibrils with consequent disappearance of the typical banding observed in control WST rat heart (Fig. 2Ab). PSELT mitigated the cardiac alteration typical of SHHF animals. Indeed, SHHF rats treated with PSELT showed improved ultrastructural features where myofibrils exhibited well organized sarcomeres, mitochondria were regularly arranged between myofibrils, and the nuclei displayed a regular form (Fig. 2Ac). We did not observe pathological ultrastructural rearrangements in the hearts of WST rats treated with PSELT (WST + PSELT) (Fig. 2Ad).

To determine whether PSELT could affect the expression levels of desmin (i.e., a key factor involved in the maintenance of cardiac cellular architecture and structure) in failing hearts, we performed western blot and relative densitometric analysis on the soluble and insoluble fractions of LV extracts of each experimental group. Figure 2B shows that in the soluble fraction of SHHF cardiac tissue, desmin expression was significantly reduced compared with that of WST hearts, while PSELT was able to restore protein expression in the SHHF model (Fig. 2B). We did not find any significant difference in desmin expression levels between WST and WST + PSELT groups. Although we observed a tendency of an increase of desmin levels in the cardiac insoluble fraction of SHHF group compared with that of WST group, as well as in that of SHHF + PSELT group with respect to that of SHHF group, these differences did not reach statistical significance (Fig. 2B).

### PSELT affects fibrosis-, senescence-, and DNA damage-related markers, and SELENOT expression in SHHF animals

To gain further mechanistic insight in the cardioprotective action of PSELT, we performed additional western blot and relative densitometric analyses on LV extracts (Fig. 2C). In particular, to determine the ability of the peptide to counteract HF-dependent cardiac remodelling and fibrosis, we assessed the gelatinolytic activity of MMPs through SDS-PAGE zymography. Our results showed that MMP-2, in its active form (63 kDa), is up-regulated in myocardial extracts of SHHF compared to WST, while we found a significant reduction of its activity in SHHF + PSELT compared to SHHF group. We did not find any significant change in MMP-2 zymographic activity between WST + PSELT and WST groups. Then, we evaluated the levels of CTGF, a well-established cardiac fibrosis marker in WST and SHHF hearts (Fig. 2C). As shown in the figure, SHHF rats exhibited increased cardiac expression levels of CTGF compared to WST rats, whereas PSELT significantly reduced CTGF levels in hearts from SHHF rats compared to that from SHHF alone (Fig. 2C). No significant difference in CTGF expression was found between WST and WST + PSELT experimental groups. To evaluate the role of PSELT in mitigating senescence and DNA damage associated with HF, we determined the expression levels of p21 and p53, and γH2AX, respectively. Results depicted in Fig. 2C indicate a significant increase of p21 and p53 expression levels in the heart of SHHF rats compared to WST hearts, and a significant reduction of these two markers in SHHF + PSELT group compared to SHHF group. Regarding p53 expression, a significant decrease was also detected in WST + PSELT group in comparison to WST group. In contrast, p21 expression did not vary among these two experimental groups (Fig. 2C). Our western blot analysis in Fig. 2C also showed that the protein expression levels of γH2AX was significantly increased in SHHF animals compared with control rats (WST group), while in SHHF rats treated with PSELT (SHHF + PSELT group) γH2AX upregulation was prevented. We did not find significant difference among WST and PSELT alone groups. Finally, we evaluated whether PSELT administration could affect the cardiac expression of endogenous SELENOT in control and failing hearts. Our results showed that SELENOT cardiac expression levels are significantly higher in SHHF rats than in WST rats (Fig. 2C), while they were significantly decreased in SHHF + PSELT group compared to the SHHF group. Exogenous PSELT also enhanced SELENOT expression under physiological conditions, as the protein expression increased in WST + PSELT group compared to WST group, even if the difference did not reach significance.

### PSELT mitigates contractile impairment and infarct size following IRI in both WST and SHHF animals

**Fig. 3:**
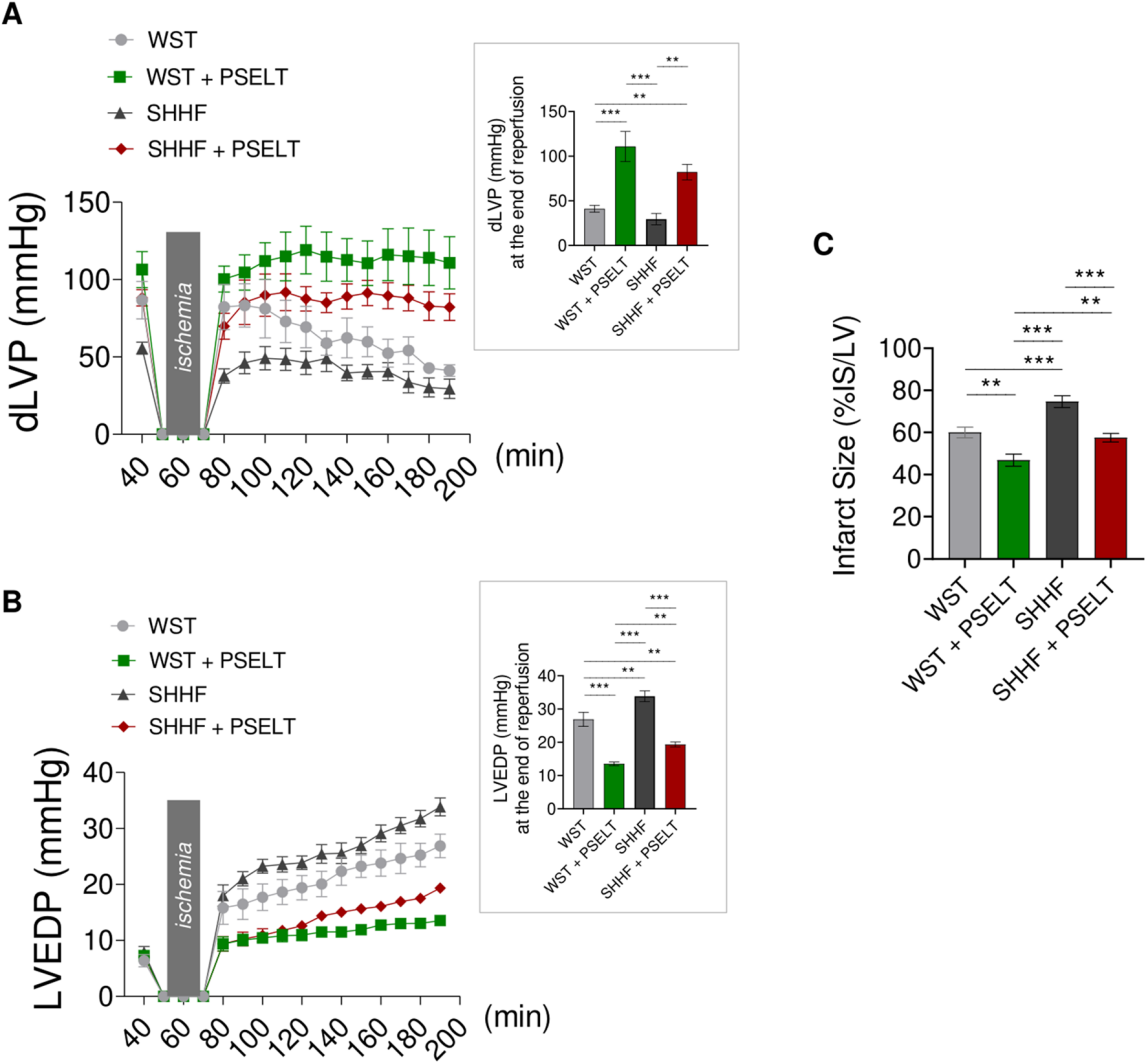
Effect of PSELT on cardiac function and infarct size (IS) of Langendorff-perfused rat hearts subjected to I/R injury. **A** dLVP and **B** LVEDP variations. Gray boxes indicate ischemic administration (Bonferroni multiple comparison test). dLVP = 21.98% of total variation between groups (*p* < *0.001*); LVEDP = 19.46% of total variation between groups (*p* < *0.001*). Inset histograms show dLVP and LVEDP at the end of reperfusion. Data are expressed as changes of dLVP and LVEDP values (millimeters of mercury, mmHg) from stabilization to the end of reperfusion with respect to the baseline values for WST, WST + PSELT, SHHF, SHHF + PSELT groups (n = 6 hearts/group). Significant differences were detected by one-way ANOVA and Newman-Keuls multiple comparison test. **p < 0.01, ***p < *0.001*. **C** Infarct size (IS) expressed as a percentage of the LV mass (% IS/LV) in WST, WST + PSELT, SHHF, SHHF + PSELT groups. Significant differences were detected by one-way ANOVA and Newman-Keuls multiple comparison test. **p < 0.01, ***p < *0.001*

To evaluate the protective effects of PSELT following IRI, and to assess the impact of the peptide on the susceptibility to myocardial infarction in WST and SHHF rats, the isolated and perfused Langendorff heart model was used and the post-ischemic systolic and diastolic recovery, together with the extent of myocardial IS was evaluated. After 40 min of stabilization (before induction of IRI), the analysis of basal cardiac parameters of all groups indicated that the heart from SHHF group had a compromised cardiac performance, as evidenced by a significant reduction of dLVP, + (LVdP/dt) max and ^_^(LVdP/dt) max, together with a higher CP compared to WST group (Table 2). We observed that PSELT was capable of significantly improving these baseline cardiac parameters in SHHF model (Table 2). To assess the effects of PSELT on post-ischemic cardiac recovery, dLVP (*i.e.,* the inotropic activity) and LVEDP values (*i.e.,* an important index of the contracture state and cardiac damage) were evaluated in the post-ischemic phase. As shown in Fig. 3A, dLVP values, during the reperfusion and at the end of reperfusion, were significantly improved in the heart of rats treated with PSELT, both in WST and SHHF animals, compared with their control counterparts treated with saline (*dLVP values at the end of reperfusion in WST group: 41* ± *4 mmHg vs WST* + *PSELT group: 111* ± *17 mmHg; SHHF group: 29* ± *6 mmHg vs SHHF* + *PSELT group: 82* ± *9 mmHg*). Regarding LVEDP parameter, Fig. 3B indicates first that cardiac contracture was more evident in SHHF rats as LVEDP was significantly higher in SHHF group than in WST group (*LVEDP value at the end of reperfusion in WST group: 27* ± *2 mmHg vs SHHF group: 34* ± *2 mmHg*), while LVEDP significantly decreased in WST and in SHHF rats exposed to PSELT compared to their control groups (*LVEDP value at the end of reperfusion in WST group: 27* ± *2 mmHg vs WST* + *PSELT group: 14* ± *1 mmHg; SHHF group: 34* ± *2 mmHg vs SHHF* + *PSELT group: 19* ± *1 mmHg*). To corroborate the cardioprotective action of PSELT, we evaluated the extent of IRI-induced IS (expressed as a percentage of LV mass), revealing that (Fig. 3C) IS significantly decreased in the heart from rats treated with PSELT with respect to those treated with saline (~ *60* ± *3% of IS/LV in WST group vs* ~ *47* ± *3% of IS/LV in WST* + *PSELT group and* ~ *75* ± *3% of IS/LV in SHHF group vs* ~ *58* ± *2% of IS/LV in SHHF* + *PSELT group*) and further increased in the heart of SHHF rats compared with that of WST rats (~ *60* ± *3% of IS/LV in WST group vs* ~ *75* ± *3% of IS/LV in SHHF group*).

**Table 2:** Basal cardiac parameters after stabilization

Experimental group	dLVP (mmHg)	LVEDP (mmHg)	+ (LVdP/dt)_max_ (mmHg/s)	−(LVdP/dt)_max_ (mmHg/s)	HR (beats/min)	CP (mmHg)
WST	87 ± 12	6 ± 1	2813 ± 191	− 1944 ± 154	247 ± 8	68 ± 1
WST + PSELT	106 ± 12	7 ± 1	3091 ± 138	− 2121 ± 62	238 ± 5	68 ± 2
SHHF	56 ± 4^*^	8 ± 1	2128 ± 121^**^	− 1580 ± 75^*^	256 ± 11	107 ± 2^***^
SHHF + PSELT	88 ± 5^†^	7 ± 1	2547 ± 93^†^	− 1873 ± 68^†^	250 ± 4	86 ± 2^†††^

dLVP: developed left ventricular pressure; LVEDP: left ventricular end-diastolic pressure; + (LVdP/dt)_max_: maximal rate of left ventricular contraction; -(LVdP/dt)_max_: maximal rate of left ventricular pressure decline; HR: heart rate; CP: coronary pressure

Basal cardiac parameters after stabilization. n = 6 for each experimental group; data are expressed as means ± SEM; ^*^p < 0.05; ^**^p < 0.01; ^***^p < *0.001* WST *vs* SHHF; ^†^*p* < *0.05;* ^†††^*p* < *0.001* SHHF *vs* SHHF + PSELT

Statistical significance: One-way ANOVA and the non-parametric Newman-Keuls Multiple Comparison Test

### PSELT counteracts ISO-induced hypertrophy in H9c2 cardiomyocytes through the Sec-containing redox catalytic site

**Fig. 4:**
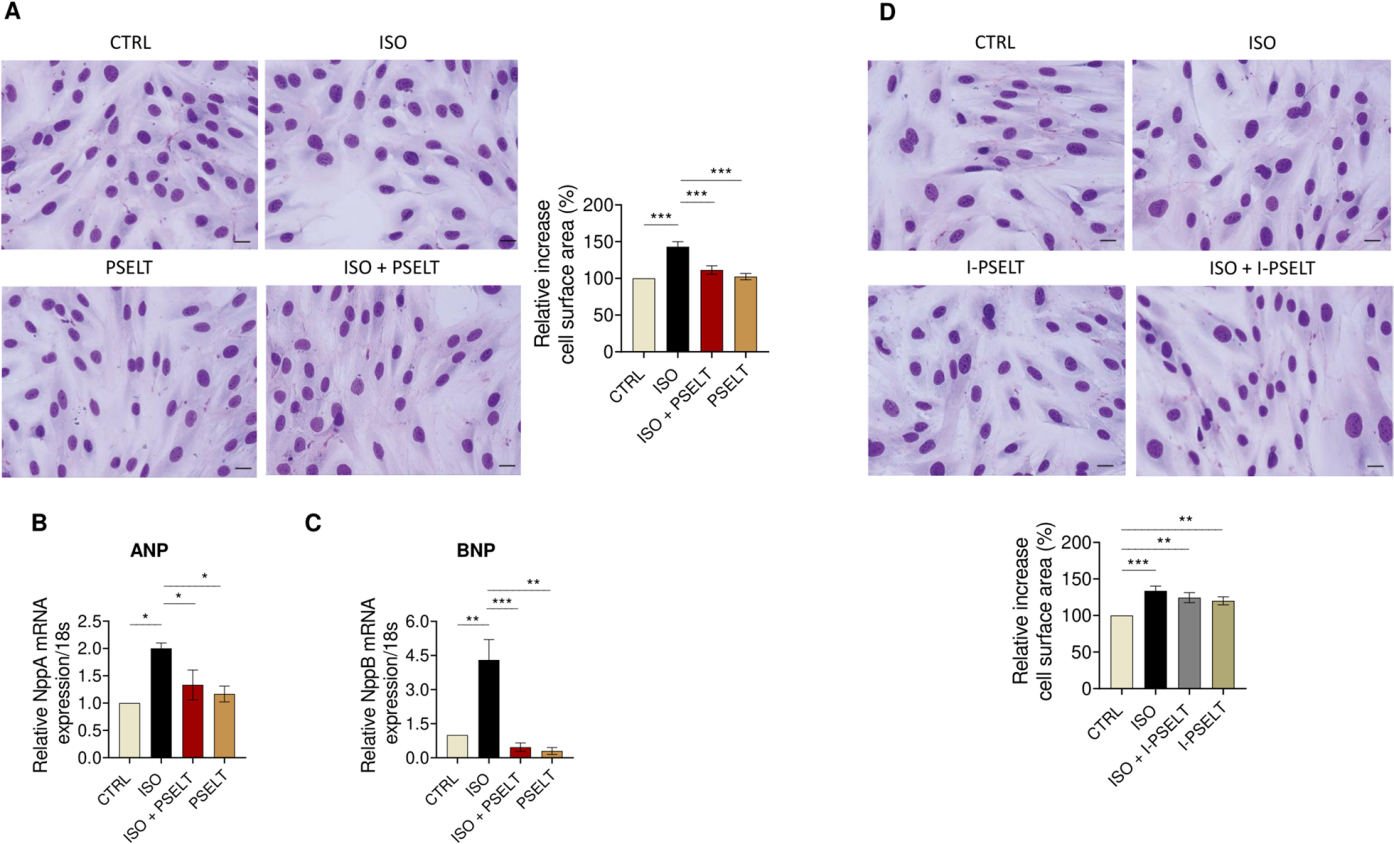
Effects of PSELT on cardiomyocytes hypertrophy induced by isoproterenol in vitro. **A** Morphological staining of H9c2 cells exposed to vehicle (saline, NaCl 0.9%) indicated as a CTRL, isoproterenol (ISO) (100 µM), ISO + PSELT and PSELT (5 nM) for 48 h and relative quantification of cell surface area performed by ImageJ. Scale bars: 25 μm. Data are expressed as mean ± SEM. Significant differences were detected by one-way ANOVA and Newman-Keuls multiple comparison test. ***p < 0.001. Evaluation of mRNA expression levels of **B** NppA (ANP) and **C** NppB (BNP), by qPCR in H9c2 cardiomyocytes exposed to vehicle (CTRL), ISO, ISO + PSELT and PSELT for 48 h. Samples were analyzed in duplicate (n = 3 independent experiments). The relative mRNA expression levels of the hypertrophic genes were normalized to 18S rRNA. Fold change is calculated on the basis of the 2^−ΔΔCT^. Data are expressed as the mean ± SEM. Significant differences were detected by one-way ANOVA and Newman-Keuls multiple comparison test. *p < 0.05; **p < 0.01, ***p < 0.001. **D** Morphological staining of H9c2 cells exposed to vehicle (saline, NaCl 0.9%) indicated as a CTRL, isoproterenol (ISO) (100 µM), ISO + I-PSELT and I-PSELT (5 nM) for 48 h and relative quantification of cell size performed by ImageJ. Scale bars: 25 μm. Data are expressed as mean ± SEM and significant differences were detected by one-way ANOVA and Newman-Keuls multiple comparison test. **p < 0.01; ***p < 0.001

We conducted an in vitro study to determine whether PSELT could mitigate ISO-dependent cardiomyocyte hypertrophy. To this aim, we exposed H9c2 cells to ISO and PSELT either separately or in co-treatment for 48 h, and a cell morphological analysis was carried out. Cell staining showed that ISO induced a significant increase in cell size compared to control cells, while PSELT significantly counteracted ISO-induced cell hypertrophy (Fig. 4A). Cells exposed to PSELT alone did not show a significant change in cell surface area. We then tested the protective action of the peptide against ISO-induced cell hypertrophy by assessing gene expression levels of key markers of the hypertrophic process. qPCR analysis revealed that the hypertrophic factors ANP (Fig. 4B) and BNP (Fig. 4C) were significantly upregulated in ISO-stimulated H9c2 cardiomyocytes compared to control cells. Conversely, mRNA expression levels of both ANP and BNP were significantly reduced in ISO + PSELT co-treated cells compared to ISO treated cells (Fig. 4B, C). In the same experimental setting, the inert form of PSELT (I-PSELT) was ineffective in mitigating ISO-mediated cell hypertrophy (Fig. 4D).

### ISO increases SELENOT protein expression in H9c2 cardiomyoblasts and does not exacerbate cell size increase in SELENOT-knockdown H9c2 cells

**Fig. 5:**
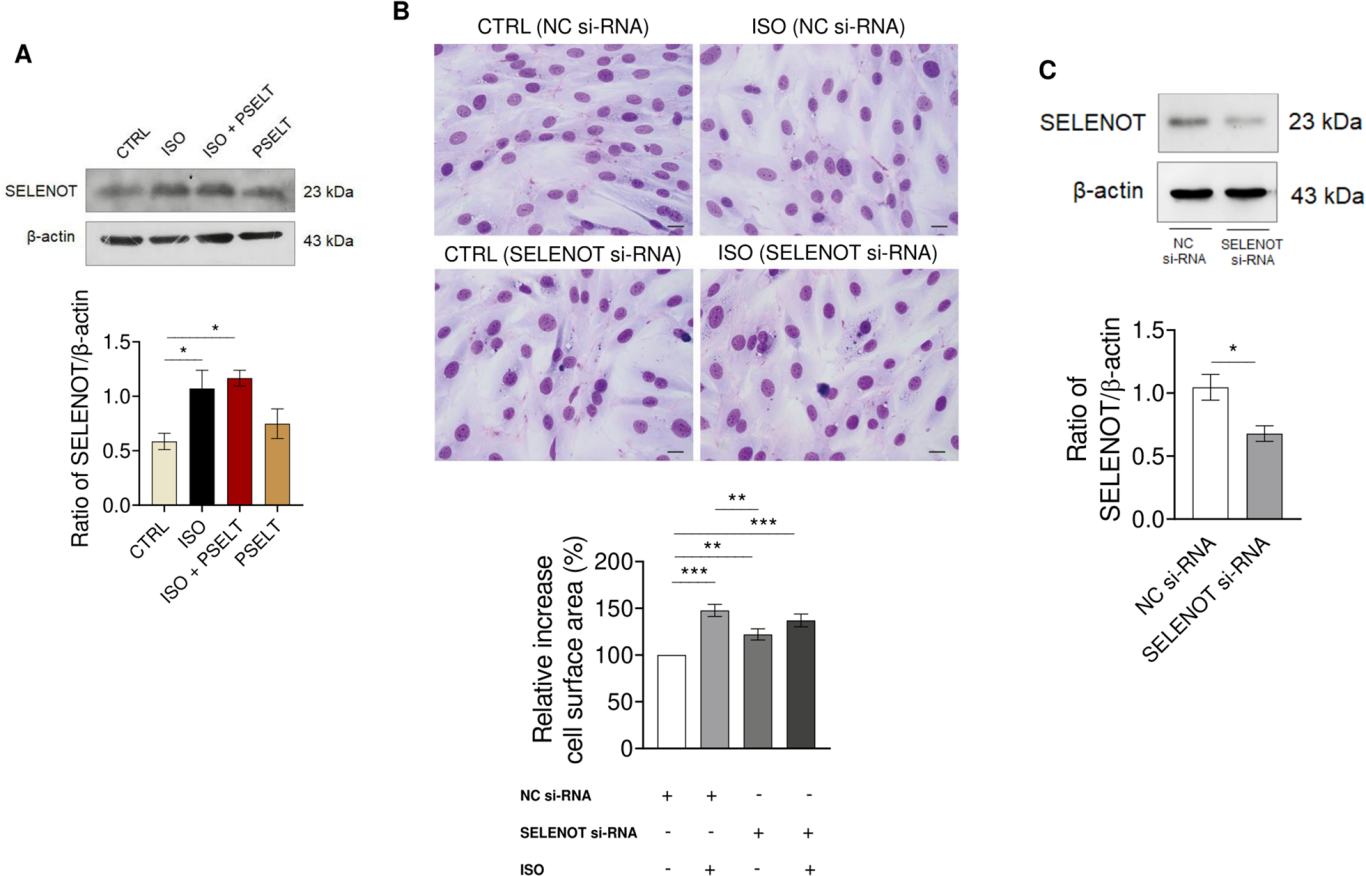
Effects of ISO and PSELT on SELENOT expression and role of endogenous protein on cell hypertrophy in H9c2 cardiomyocytes. **A** Western blot analysis of SELENOT expression in H9c2 cells exposed to vehicle (saline, NaCl 0.9%) indicated as a CTRL, isoproterenol (ISO) (100 µM), ISO + PSELT and PSELT (5 nM). Histograms represent the ratio of densitometric analysis of protein:loading control. Data are expressed as the mean ± SEM (n = 3 independent experiments). Significant differences were detected by one-way ANOVA and Newman-Keuls multiple comparison test. *p < 0.05. **B** Morphological staining of H9c2 cells transfected with Negative control si-RNA (NC si-RNA) or Selenoprotein T si-RNA (SELENOT si-RNA) for 36 h and then treated with or without ISO (100 µM) for additional 48 h. Scale bars: 25 μm. Cell surface area (%) was quantified by ImageJ. Data are expressed as mean ± SEM from three independent experiments. Significant differences were detected by one-way ANOVA and Newman-Keuls multiple comparison test. **p < 0.01; ***p < 0.001. **C** Representative Western blot analysis of SELENOT in H9c2 cardiomyocytes transfected with NC si-RNA or SELENOT si-RNA for 36 h. Histograms represent the ratio of densitometric analysis of protein:loading control. Data are expressed as the mean ± SEM (n = 3 independent experiments). Significant differences were detected by t-test. *p < 0.05

To investigate whether ISO could affect SELENOT levels in cardiomyocytes, we treated H9c2 cells with ISO for 48 h in the presence or absence of PSELT, and performed western blot analysis. As shown in Fig. 5A, the treatment with ISO affected SELENOT levels, which were significantly increased in ISO and ISO + PSELT-treated cells compared to control cells (Fig. 5A). PSELT treatment slightly increased SELENOT levels as observed in ISO + PSELT-treated cells *vs* ISO-treated cells and PSELT-treated cells *vs* control cells, although the difference was not statistically significant. To determine the role of endogenous SELENOT in the hypertrophic response induced by ISO, we evaluated whether ISO could exert a stronger effect (in terms of cell size increase) when SELENOT was silenced. To this aim, we transfected H9c2 cells with si-NC or SELENOT-siRNA and exposed them to ISO or vehicle. Morphological staining and relative quantification of the cell surface area shown in Fig. 5B revealed that ISO induces a similar significant increase of cell surface area in both si-NC cells and SELENOT knockdown cells, compared to si-NC cells treated with vehicle. We also found increased cell size in SELENOT knockdown cells treated with vehicle, compared to si-NC cells. The effectiveness of si-SELENOT gene silencing is shown by the significant decrease of SELENOT protein level in SELENOT knockdown cells compared to control (si-NC) (Fig. 5C).

### PSELT counteracts ISO-induced hypertrophy in AC16 human ventricular cardiomyocytes through the Sec-containing redox catalytic site

**Fig. 6:**
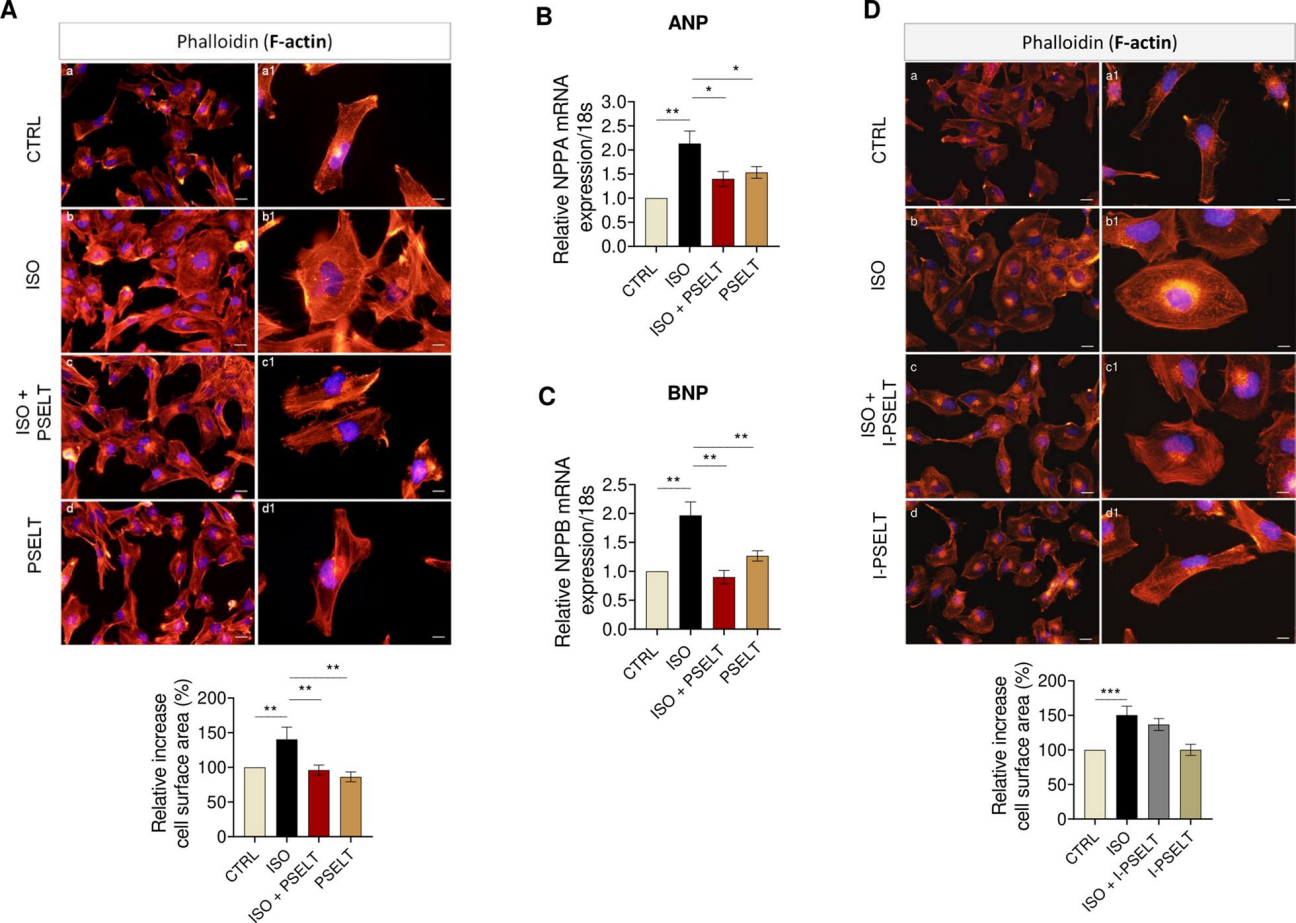
Effects of PSELT against cell hypertrophy induced by isoproterenol in vitro. **A** F-actinin morphological staining of AC16 human cardiomyocytes exposed to vehicle (saline, NaCl 0.9%) indicated as a CTRL, isoproterenol (ISO) (100 µM), ISO + PSELT and PSELT (5 nM) for 48 h and relative quantification of cell surface area performed by ImageJ. *Scale bars*: (a, b, c, d) 25 μm and (a1, b1, c1, d1) 12.5 μm. Data are expressed as mean ± SEM. Significant differences were detected by one-way ANOVA and Newman-Keuls multiple comparison test. **p < 0.01. Evaluation of mRNA expression levels of **B** NPPA (ANP) and **C** NPPB (BNP), by qPCR in AC16 human cardiomyocytes exposed to vehicle (CTRL), ISO, ISO + PSELT and PSELT for 48 h. Samples were analyzed in duplicate (n = 3 independent experiments). The relative mRNA expression levels of the hypertrophic genes were normalized to 18S rRNA. Fold change is calculated on the basis of the 2^−ΔΔCT^. Data are expressed as the mean ± SEM. Significant differences were detected by one-way ANOVA and Newman-Keuls multiple comparison test. *p < 0.05; **p < 0.01. **D** F-actinin staining of AC16 cells exposed to vehicle (saline, NaCl 0.9%) indicated as a CTRL, isoproterenol (ISO) (100 µM), ISO + I-PSELT and I-PSELT (5 nM) for 48 h and relative quantification of cell size performed by ImageJ. Scale bars: (a, b, c, d) 25 μm and (a1, b1, c1, d1) 12.5 μm. Data are expressed as mean ± SEM and significant differences were detected by one-way ANOVA and Newman-Keuls multiple comparison test. ***p < 0.001

We also assessed the protective action of PSELT against ISO-induced hypertrophy in human adult ventricular cardiomyocytes using AC16 cells exposed to ISO and PSELT alone or in co-treatment for 48 h, and evaluating the cell size and the mRNA levels of ANP and BNP. As shown in Fig. 6A, ISO induced a significant increase in cell surface area and upregulated both ANP (Fig. 6B) and BNP (Fig. 6C) expression levels compared to control cells, while PSELT significantly mitigated ISO-induced cell hypertrophy and increased ANP and BNP levels. AC16 cardiomyocytes exposed to PSELT alone did not show significant changes neither in cell surface area nor in ANP and BNP gene expression (Fig. 6A–C). Furthermore, the treatment with the inert form of PSELT (I-PSELT) in the same experimental condition was ineffective in counteracting ISO-dependent cell hypertrophy (Fig. 6D).

### Chronic ISO exposure upregulates intracellular SELENOT and triggers its secretion in AC16 human ventricular cardiomyocytes

**Fig. 7:**
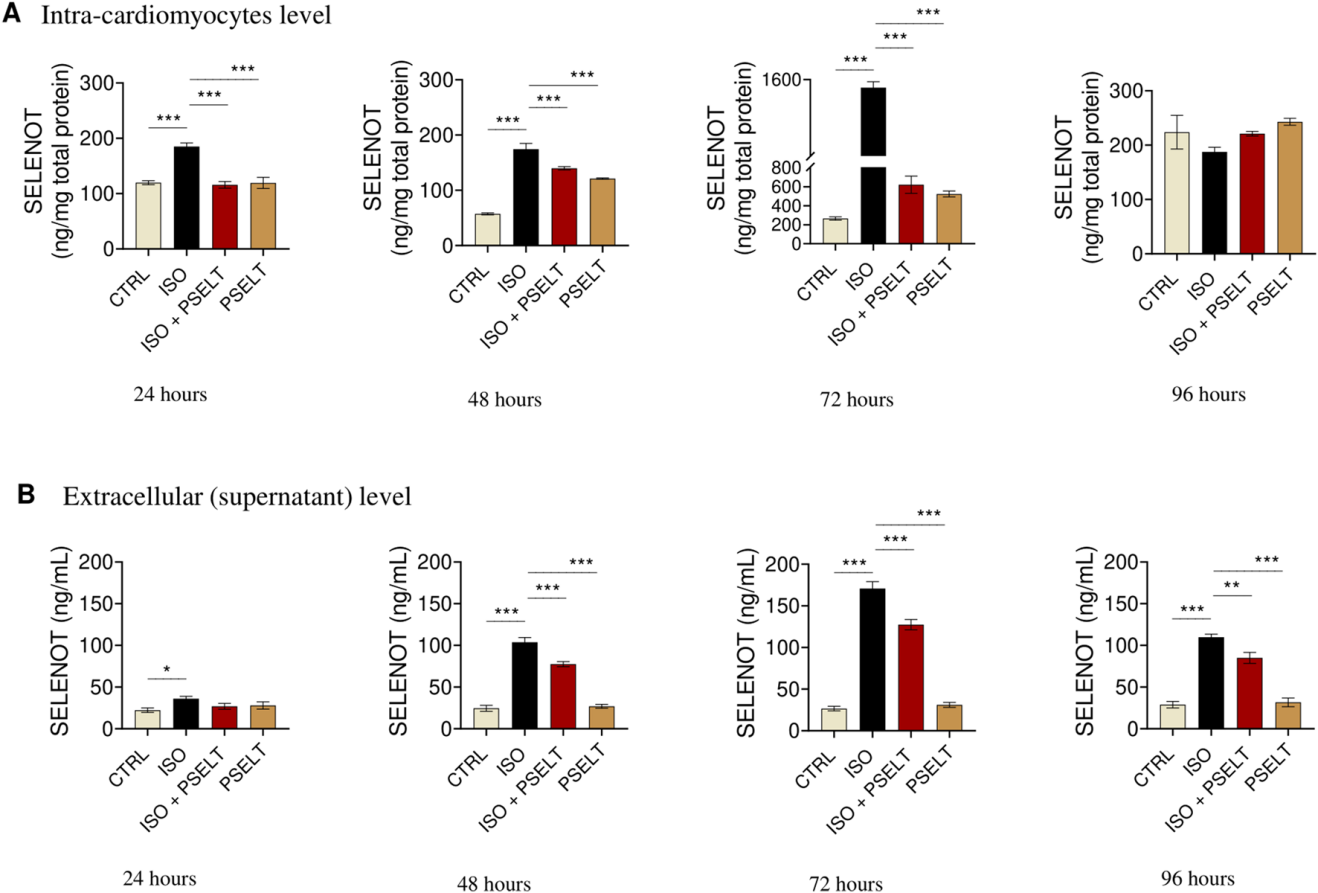
Effects of isoproterenol (ISO) and PSELT on Selenoprotein T (SELENOT) intracellular and extracellular levels in human cardiomyocytes. **A** Intra-cardiomyocyte levels of SELENOT in AC16 cells exposed to vehicle (saline, NaCl 0.9%) indicated as a CTRL, isoproterenol (ISO) (100 µM), ISO + PSELT and PSELT (5 nM) for 24–48-72–96 h. **B** SELENOT levels in cell culture medium (supernatant) of AC16 cardiomyocytes exposed to vehicle (saline, NaCl 0.9%) indicated as a CTRL, isoproterenol (ISO) (100 µM), ISO + PSELT and PSELT (5 nM) for 24–48-72–96 h. Data are expressed as means ± SEM. Significant differences were detected by one-way ANOVA and Newman-Keuls multiple comparison test. *p < 0.05; **p < 0.01; ***p < 0.001

To evaluate whether ISO could affect the intracellular and extracellular levels of SELENOT in human cardiomyocytes following chronic and very prolonged hypertrophic stimulus, and investigate the role of PSELT in this process, we quantified SELENOT levels in cell lysate and cell supernatant of AC16 cells exposed to ISO and PSELT alone or in co-treatment for 24, 48, 72 and 96 h. Our results showed that ISO significantly increased, in a similar manner, SELENOT intracellular levels at 24 h and 48 h compared to control cells; this increase was much more evident at 72 h, while no significant variations were observed among groups after 96 h of ISO exposure (Fig. 7A). Conversely, PSELT significantly reduced the increase in intracellular expression of SELENOT after ISO treatment at 24, 48, and 72 h compared with ISO alone (Fig. 7A). Figure 7B, representing SELENOT levels in the cell culture medium, showed that ISO triggers SELENOT secretion in a time-dependent manner up to 72 h, since extracellular protein levels were significantly higher in ISO group at 24, 48, and 72 h compared to control group. This trend was maintained among groups at 96 h. Conversely, compared with ISO alone, PSELT treatment significantly decreased SELENOT levels after ISO treatment at all the exposure times (Fig. 7B).

### PSELT mitigates ISO-dependent ultrastructural abnormalities in AC16 human ventricular cardiomyocytes

**Fig. 8:**
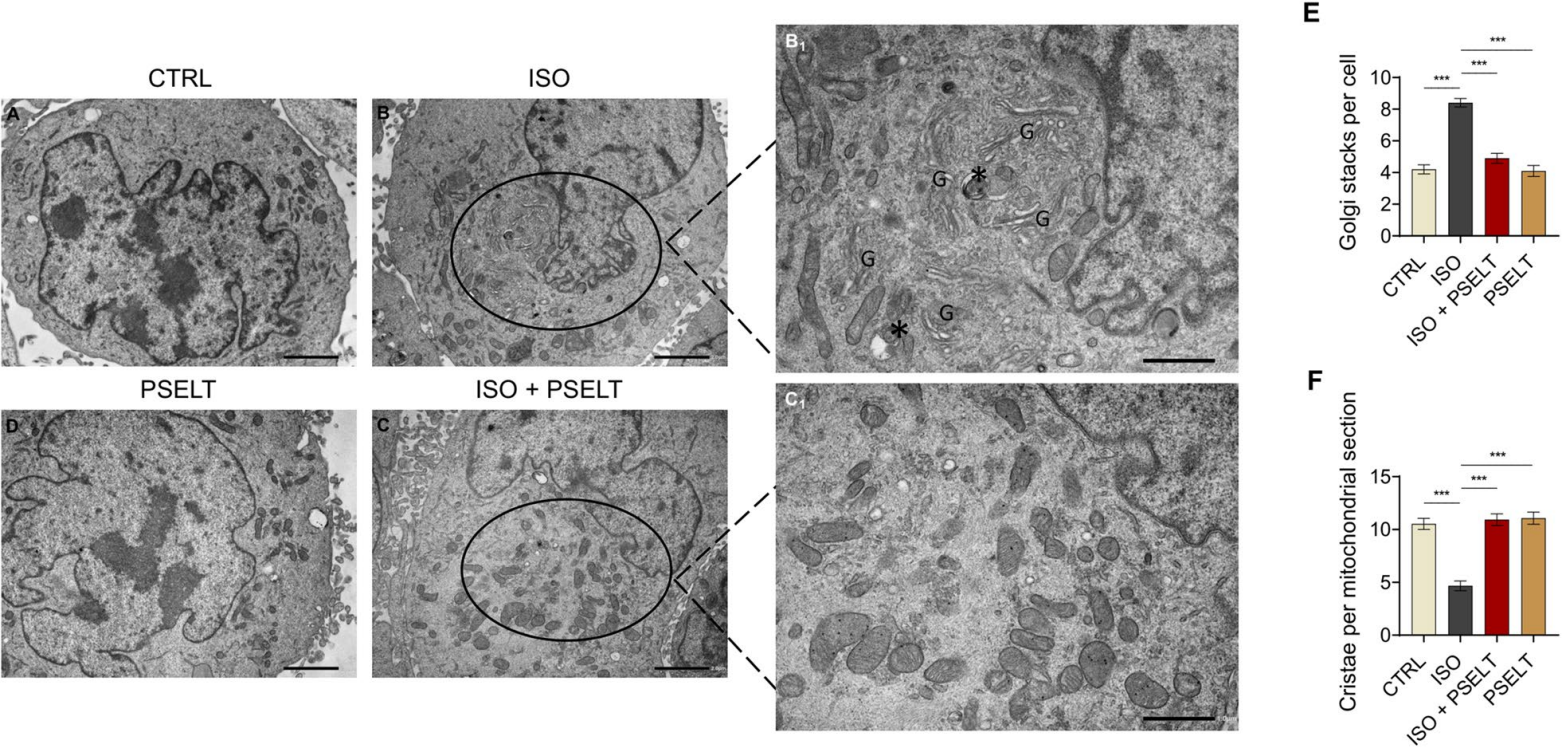
Ultrastructure representations obtained by transmission electron microscopy (TEM) on AC16 human cardiomyocytes exposed to ISO and PSELT, alone or in combination. **A** Control group (scale bar: 2 μm, magnification × 4000); **B** ISO group (scale bar: 2 μm, magnification × 4000) and **B**_**1**_ (scale bar: 1 μm, magnification × 8000; **C** ISO + PSELT group (scale bar: 2 μm, magnification × 4000) and **C**_**1**_ (scale bar: 1 μm, magnification × 8000; **D** PSELT group (scale bar: 2 μm, magnification × 4000); **E** quantitative TEM analysis showing the number of Golgi stacks per cell that was determined by a counting grid method from 10 different electron microscope images for each sample; **F** quantitative TEM analysis of mitochondrial cristae number based on the quantification of the cristae per mitochondrial section from different mitochondria. Only mitochondria with intact and completely visible borders have been included in the analysis

Through TEM analyses, we focused on the potential protective action of PSELT against ISO-induced ultrastructural alterations in AC16 cardiomyocytes. Our results revealed normal ultrastructure features in control cardiomyocytes, with intact membranes and a homogeneous cytoplasm of moderate electron density filled with mitochondria, endoplasmic reticulum (ER), and a moderately developed Golgi apparatus (Fig. 8A). Conversely, human cardiomyocytes exposed to ISO showed accumulation of autophagic vacuoles and a marked hyperplasia (expansion) of the Golgi apparatus, accompanied by increased vacuolation of the adjacent cytoplasm (Fig. 8B); some mitochondria also showed a reduction in the number of cristae, and a rarefied matrix (Fig. 8B**1**). Conversely, cardiomyocytes exposed to ISO + PSELT exhibited a remarkable restoration of conventional cardiomyocyte ultrastructure (Fig. 8C). Indeed, in ISO + PSELT-treated cells mitochondria appeared well-preserved, the autophagic vacuoles decreased and the Golgi compartments generally exhibited reduced size. Moreover, the number of the vacuoles associated with the Golgi complex were reduced compared with ISO-treated cells (Fig. 8C1). PSELT treatment alone did not induce significant changes in the structural and subcellular organization of cardiomyocytes (Fig. 8D). The quantitative assessment estimating the hyperplasia of the Golgi apparatus showed that the number of Golgi stacks per cell was significantly higher in ISO group compared to control group, while they significantly decreased in ISO + PSELT group with respect to ISO group (Fig. 8E). The quantitative analysis evaluating mitochondrial cristae through quantifying the cristae per mitochondrial section from different mitochondria showed an opposite trend; in particular, ISO reduced the mitochondrial cristae number compared to control cells; conversely, PSELT treatment was able to prevent ISO-dependent reduction in mitochondrial cristae number (Fig. 8F). No significant difference in the number of Golgi stacks per cell and in the mitochondrial cristae number have been detected between control and PSELT groups (Fig. 8E, F).

## Discussion

Despite the progress of pharmacological and nonpharmacological therapy in limiting and/or reversing the signs and symptoms of HF, mortality and morbidity related to this multifactorial syndrome remain elevated. Thus, identifying additional strategies to support earlier detection and treatment and deciphering key pathophysiological pathways to be targeted for achieving maximal clinical benefit in patients with HF still represents a critical medical issue [11, 46]. The key role of the oxidoreductase SELENOT in cardiomyocyte differentiation and acute cardioprotection [19, 21, 22] pointed out this selenoprotein as an emerging regulator of cardiac development and function while raising an important unaddressed point concerning the beneficial action of this protein in chronic settings of cardiac dysfunction. In dealing with this point, here we provided evidence that SELENOT may exert beneficial effects in a well-consolidated preclinical model of HF at both cardiac and systemic levels, by targeting key pathways that are disrupted in HF, including inflammation, fibrosis and susceptibility to IRI. On the other hand, we showed that SELENOT can prevent ISO-induced hypertrophy in rat and human cardiomyocytes, where the protein may also act as stress-sensor agent during the hypertrophic response, pointing out a novel role of this selenoprotein in the pathophysiology of HF.

Large evidence recognized that, in human HF, inflammation plays a major pathophysiological role that affects clinical outcome [47–49]. Similarly, increased circulating cytokines are reported in preclinical models of HF, and their levels correlate with age advancement, systemic congestion, and disease progression. This is also evident in SHHF rat, that represents a consolidated model to examine the cellular and molecular bases of hypertension-induced cardiac hypertrophy and subsequent HF, being able to recapitulate key systemic and cardiac functional and structural alterations documented in patients [13]. Here, we employed SHHF rats at 15 month of life (*i.e.,* time at which animals exhibit significant systemic alteration and impaired cardiac function) [24, 25] to assess the systemic and cardioprotective role of SELENOT, by exploiting its small mimetic peptide (PSELT) that includes the redox active site (with the CVSU motif) of the full protein and that affords cardioprotection and neuroprotection intracellularly as a cell-penetrating peptide [21, 50]. The advantages in using this small peptide lies in the fact that the biological activity of SELENOT strictly depends on the CVSU motif, which interacts with other cellular components through redox reactions. Moreover, because of their distinct mechanism of action, small peptides may represent a more suitable strategy in the translational/clinical scenario than conventional therapies [51].

Our results indicate that PSELT displayed both systemic and direct anti-inflammatory actions in SHHF rats (by reducing the circulating levels of IL-1β and TNF-α) and LPS-stimulated RAW 264.7 macrophages [by reducing CD80 expression, a hallmark of M1-like proinflammatory phenotype [52, 53], suggesting that the peptide may relieve macrophage M1 activation—and the consequent release of pro-inflammatory cytokines—that closely correlate with the occurrence and progression of HF [54]. Accordingly, a large body of evidence indicates that macrophages infiltrate the heart during the early stages of heart disease, contributing to adverse cardiac remodeling and HF [55]. On the other hand, PSELT decreased plasma levels of LDH, an important indicator of myocardial injury [56] and BNP, a selective indicator of heart damage severity [57]. We also found that PSELT reduced myocardial production of GAL-3, a β-galactoside–binding lectin, whose plasma and myocardial levels can reflect the progression of LV fibrosis and cardiomyopathy, and which represents a diagnostic and prognostic marker in both acute and chronic HF, particularly when assessed together with N-terminal pro–BNP (NT-proBNP) [58, 59]. To gain insight into the mechanistic action behind the protective effect of PSELT, we found that the severe ultrastructural alterations typical of SHHF hearts leading to myofibrillar degeneration were markedly mitigated by the peptide. Based on this data, we evaluated the effect of PSELT on the expression levels of desmin, the major protein component of the intermediate filaments of the cytoskeleton in muscle (including cardiac) cells [60]. We found a marked reduction of total desmin levels in the soluble fraction of SHHF hearts, indicating a decreased intact desmin in the failing heart that likely reflects, at least in part, protein aggregates accumulation in the insoluble fraction [61, 62]. However, we did not find significant modulation of total desmin in insoluble fraction in our HF model. It is interesting to note that PSELT rescued total desmin levels in the soluble fraction of failing hearts. Together with TEM findings, this suggested that the peptide may preserve the structure of intact desmin and protect the structural integrity of the contractile apparatus of cardiomyocytes, thus limiting cardiac dysfunction.

Compelling evidence indicates a higher incidence and morbidity of HF with advanced age, since aged cardiomyocytes gain a senescent phenotype characterized by cellular hypertrophy, interstitial fibrosis and oxidative stress [63, 64]. In this regard, we analysed the effect of PSELT on important mediators of fibrosis and aging in SHHF rats, which at 15-month-old exhibit not only overt HF, but also a senescent phenotype, representing a key model to study the synergistic effects of aging and HF on myocardial alterations [65]. Particularly, we evaluated the cardiac activity of MMP-2, a biomarker in patients with HF with a key role in cardiac remodelling [66] and the cardiac expression levels of CTGF, the main regulator of fibrosis during maladaptive ventricular remodeling and HF progression also acting as downstream messenger of GAL-3 [67, 68]; we then assessed the cardiac expression levels of p21 and p53, indicators of cardiac aging that mediate senescence in response to stressors [69], and γH2AX, a sensitive indicator of DNA damage and integrity of the repair mechanism [70]. Interestingly, PSELT prevented the increased activity of MMP-2 and reduced the levels of all the other markers in SHHF hearts, indicating that the peptide can exert beneficial action also at cardiac level by mitigating fibrosis and aging-associated cardiac impairment. Notably, in HF rats PSELT also prevented the upregulation of myocardial γH2AX, whose levels increased in failing hearts as revealed by both experimental and human studies [71, 72], suggesting a role of the peptide in protecting the damaged heart against cellular DNA damage. Further, in investigating the influence of PSELT on the cardiac expression of endogenous SELENOT, we found that in the SHHF model the protein levels were markedly increased, suggesting that the failing heart may upregulate this important antioxidant selenoprotein as a part of a protective response to limit cellular damage during a chronic myocardial insult. This is reminiscent of the cardiac profile of key redox-active selenoproteins whose increased mRNA and protein levels associate with their ability to counteract oxidative damage in rodent models of LV hypertrophy and HF [73, 74]. Intriguingly, PSELT strongly reduced SELENOT levels in failing hearts, indicating that the exogenous peptide can exert itself a protective action by also re-establishing SELENOT levels to a normal condition.

The role of PSELT in preserving cardiac ultrastructure and reducing key biomarkers of HF prompted us to extend the cardioprotective study, by subjecting the hearts of control and SHHF rats previously treated with PSELT to IRI, and evaluate hemodynamic parameters by Langendorff assay. This allowed us to examine the role of PSELT in the heart with basal dysfunction exacerbated by ischemic damage and to assess its ability to improve cardiac recovery from IRI ex vivo. In evaluating basal myocardial performance before IRI, we first found that PSELT improved contractile impairment and coronary function in SHHF model, suggesting that the peptide can ameliorate myocardial function at baseline. This view was then supported by the results obtained in the post-ischemic phase, as PSELT protected the hearts from IRI, improving contractile recovery, preventing excessive cardiac contracture, and reducing the extension of IS (*i.e.,* a key determinant of clinical outcome in post-ischemic HF patients) in both control and SHHF animals. This indicated that the chronic administration of PSELT may reduce IRI-induced adverse myocardial function in normal and failing hearts.

In deciphering the effect of PSELT on the cardiomyocyte component, we then exposed H9c2 cardiomyocytes to ISO, widely used to induce adrenergic overstimulation that drives the pathogenesis of maladaptive LV, and model hypertrophic phenotype in vitro and in vivo [75–77]. Our study showed that ISO increased cell surface area in cardiomyocytes, indicating that the in vitro model of ISO-induced cell hypertrophy was successfully established and that PSELT, but not its inert counterpart lacking Sec in the redox motif (I-PSELT), reversed ISO-provoked morphological alterations. It also decreased the gene expression of ANP and BNP, whose levels increase in response to cardiomyocyte stress during hypertrophy and HF [78]. These findings indicated that PSELT could counteract cardiomyocyte hypertrophy through its redox active site, highlighting the essential role of Sec in mediating cytoprotection.

Interestingly, as observed in SHHF heart, we also found increased SELENOT expression levels in H9c2 cells during ISO exposure. This reinforced the hypothesis that, at the cellular level, cardiomyocytes could activate an adaptive response to counteract the adrenergic overstimulation in which SELENOT may contribute, at least in part, resulting in cell protection. We therefore wondered whether endogenous SELENOT could be involved in ISO-induced hypertrophic response. To this aim, we evaluated the possibility that ISO effects (in terms of cell size increase) could be exacerbated when SELENOT was silenced. However, SELENOT knockdown did not worsen ISO-dependent cell hypertrophy, suggesting that the endogenous protein is not directly involved in the hypertrophic response of ISO; nevertheless, its deficiency increased cell surface area, indicating that this protein may be required for maintaining physiological morphology and survival of cardiomyocyte.

To move towards translatability, we employed human AC16 cardiomyocyte, a cell line widely used for assessing cardiomyocyte structure and function under normal and pathological conditions [38, 79]. After exposing AC16 cells to ISO, we found that the increased cell surface area and the elevated ANP and BNP gene expression were counteracted by PSELT, while I-PSELT was ineffective. This suggested that, also in human cells, the peptide is active in mitigating cardiomyocyte hypertrophy through its redox motif.

Based on the upregulation of SELENOT observed in failing hearts and ISO-stimulated rat cardiomyocytes, we wondered whether SELENOT could be released under hypertrophic stimulus. For this reason, we quantified the levels of the protein in human cardiomyocyte treated with ISO at increasing times (24 h-96 h) at both intracellular and extracellular levels. We observed that the increased intracellular levels of SELENOT detected after ISO treatment, that was similar at 24 h and 48 h and much more evident at 72 h (but not at 96 h), were decreased by PSELT at all these times. This reinforced the hypothesis that the hypertrophic response triggers the intracellular production of SELENOT over time and confirmed the effectiveness of PSELT. Intriguingly, SELENOT was secreted linearly with ISO exposure time, likely reflecting the extent of cell damage, while PSELT mitigated SELENOT secretion. Based on this finding, it is possible to speculate that one of the mechanisms by which cardiomyocytes respond to chronic adrenergic stimulation is SELENOT production and secretion. Although the mechanisms behind this response remain a matter of analysis, these findings may further delineate SELENOT as a potential sensor protein in stressed cardiomyocytes and provide first evidence on its potential biomarker role in the hypertrophic heart/heart disease.

Lastly, ultrastructural abnormalities of cardiomyocyte microarchitecture, including membrane integrity degeneration, Golgi hyperactivity, and reduced mitochondrial cristae number in cells exposed to ISO were reverted by PSELT. It is interesting to note that the beneficial action of PSELT may depend on its capability to act intracellularly, as reported in our previous studies [21, 50]. Whether the exogenous peptide, by penetrating cell membranes, localizes in the ER (*i.e.,* the physiological subcellular localization of endogenous SELENOT) where SELENOT crucially participates in the regulation of ER proteostasis and co-translational/post-translational protein modifications, and prevents alterations of ER ultrastructure and network in stressed cardiomyocytes [17, 18, 21, 22], remains to be established. This should be regarded with particular interest since the potential synergistic cooperation between the exogenous PSELT and the endogenous SELENOT may participate to cardiomyocyte homeostasis in physiological conditions and pathological contexts, such as adrenergic overstimulation.

## Data Availability

The data supporting the findings of this study are available within the article. All other supporting data are available from the corresponding author on reasonable request.

## Abbreviations

BNP: Brain natriuretic peptide
BSA: Bovine serum albumin
DAPI: 4′,6-Diamidino-2-phenylindole
dLVP: Developed Left ventricle pressure
DMEM-F12: Dulbecco’s Modified Eagle Medium F-12
ER: Endoplasmic reticulum
FBS: Fetal bovine serum
GAL-3: Galectin-3
HCM: Hypertrophic cardiomyopathy
HF: Heart failure
I/R: Ischemia/Reperfusion
IRI: I/R injury
IL-1β: Interleukin-1β
I-PSELT: Inert-PSELT
IS: Infarct size
ISO: Isoproterenol
KH: Krebs Henseleit
LDH: Lactate dehydrogenase
LV: Left ventricle
LVEDP: Left ventricle end-diastolic pressure
MI: Myocardial infarction
NP: Non-perfused
NADH: β-Nicotinamide adenine dinucleotide
P/S: Penicillin/streptomycin
PSELT: SELENOT-derived peptide 43–52
SELENOT: Selenoprotein T
SHHF: Spontaneously hypertensive heart failure rat
si-NC: Negative control si-RNA
siRNA: Small interfering RNA
TNF-α: Tumor necrosis factor-α
β-AR: β-Adrenergic receptor
